# A Long-Lived Family

**Published:** 1896-04

**Authors:** 


### A Long-lived Family.

A family named Hindale is reported from Mount Holly, N. T., consisting of eight members. The oldest is ninety-one years of age, and the youngest seventy-two. Their average is eighty-two years. The most remarkable feature about this family is that they are children of consumptives. The father died of that disease at forty-eight, and the mother at fifty-two. No trace of the disease is discoverable in the children.

Once this would have been inexplicable, but we now know consumption as a contagion may be communicated to those having no predisposition. This is probably the true explanation, or else on the principle of atavism, the children have inherited the constitution of a more remote ancestor.—*Insurance Monitor.*

